# The value of audiovisual sexual stimulation with virtual reality in diagnosing erectile dysfunction

**DOI:** 10.1093/sexmed/qfae014

**Published:** 2024-03-17

**Authors:** Yan-Ping Huang, Tao Jing, Kai-Feng Liu, Wei Liu, Ming Zhang, Da-Chuan Zhong, Yi-Dong Liu, Shi-Ran Xu, Mu-Jun Lu

**Affiliations:** Department of Urology and Andrology, Renji Hospital, Shanghai Jiao Tong University School of Medicine, Shanghai Institute of Andrology, Shanghai, 200001, China; Department of Urology and Andrology, The Affiliated Hospital of Qingdao University, Qingdao, 266003, China; Department of Andrology, Northern Jiangsu People's Hospital, Clinical Medical School, Yangzhou University, Affliated Hospital to Yangzhou University, Yangzhou, 225009, China; Department of Urology and Andrology, Renji Hospital, Shanghai Jiao Tong University School of Medicine, Shanghai Institute of Andrology, Shanghai, 200001, China; Department of Urology and Andrology, Renji Hospital, Shanghai Jiao Tong University School of Medicine, Shanghai Institute of Andrology, Shanghai, 200001, China; Department of Urology and Andrology, Renji Hospital, Shanghai Jiao Tong University School of Medicine, Shanghai Institute of Andrology, Shanghai, 200001, China; Department of Urology and Andrology, Renji Hospital, Shanghai Jiao Tong University School of Medicine, Shanghai Institute of Andrology, Shanghai, 200001, China; Department of Urology and Andrology, Renji Hospital, Shanghai Jiao Tong University School of Medicine, Shanghai Institute of Andrology, Shanghai, 200001, China; Department of Urology and Andrology, Renji Hospital, Shanghai Jiao Tong University School of Medicine, Shanghai Institute of Andrology, Shanghai, 200001, China

**Keywords:** audiovisual sexual stimulation, erectile dysfunction, virtual reality immersion, average erectile rigidity, effective erectile duration, diagnostic criteria

## Abstract

**Background:**

The traditional audiovisual sexual stimulation (AVSS) test may experience limitations including low erectile response rate and lack of unified diagnostic criteria.

**Aim:**

We aimed to explore the clinical value of AVSS with virtual reality (VR-AVSS) test in assessing erectile function and diagnosing erectile dysfunction (ED).

**Methods:**

Participants 18 to 60 years of age were screened for analysis in 3 clinical centers from June 2020 to March 2022. Demographic data, 5-item International Index of Erectile Function (IIEF-5), erectile hardness score (EHS), and self-reported symptom questions were collected. The ED patients and control patients were confirmed according to the IIEF-5 and EHS. All subjects watched a 60-minute erotic video by VR device during RigiScan recording. The parameters including tip average rigidity, tip effective erectile duration (duration of rigidity ≥60%, tip effective erectile duration), base average rigidity, and base effective erectile duration were evaluated.

**Outcomes:**

The main outcome of interest was the application of VR immersion technology to improve the traditional AVSS test.

**Results:**

A total of 301 ED cases and 100 eligible control patients were included for final analysis. Compared with control patients, ED cases had significantly lower IIEF-5 scores, EHS, positive response rate, and erectile rigidity and duration. The positive response rate of ED and control patients were 75.5% and 90.9%, respectively. The cutoff points of tip average rigidity, tip effective erectile duration, base average rigidity, and base effective erectile duration were 40.5% (sensitivity: 77.6%, specificity: 70.2%; *P* < .001), 4.75 minutes (sensitivity: 75.9%, specificity: 75.4%; *P* < .001), 48.5% (sensitivity: 77.6%, specificity: 75.1%; *P* < .001), and 7.75 minutes (sensitivity: 79.3%, specificity: 75.7%; *P* < .001).

**Clinical Implications:**

The technological superiority of VR will enable the VR-AVSS immersion test to be a more accurate detection than traditional AVSS modes.

**Strengths and Limitations:**

Our study applied VR immersion technology to establish the standard operation procedure for the AVSS test, which could effectively reduce the interference of adverse factors and minimize the detecting errors. However, the test data only included positive response subjects, so the true erectile status of men with a negative response to the AVSS test cannot be obtained.

**Conclusions:**

The VR-AVSS test can effectively improve the diagnostic accuracy of ED. The average rigidity and effective erectile duration were the optimal diagnostic parameters for excluding ED.

## Introduction

Erectile dysfunction (ED) is a common disease in men, and the prevalence of ED ranges from 6% to 64% in 40- to 80-year-old men.^1^^,^^2^ According to the diagnostic classification, ED can be divided into 3 types: psychological, organic, and mixed.^3^ Although the current detection methods have made great progress, there are still some difficulties in distinguishing psychological and organic ED clinically. Generally, penile erections are divided into 3 types: psychogenic erection, reflex erection, and spontaneous nocturnal erection.^4^ Psychogenic erection is triggered by auditory, visual, fantasy, or nongenital tactile stimulation that involves cortical processing of the stimulus as erotic.^5^ Penile reflex erection is triggered by tactile stimulation of the genitals via a reflex arc through the autonomic nuclei to the cavernous nerves.^5^ Nocturnal erection refers to the penile tumescence during nocturnal sleep, which mainly occurs during rapid eye movement in healthy men from infancy to the elderly.^6^ The different erection modes of penis enable clinicians have the opportunity to detect the penile erection ability and distinguish organic and psychogenic ED. Currently, nocturnal penile tumescence and rigidity (NPTR) monitoring has been recognized as an international reliable method to differentiate psychogenic ED from organic ED, and the diagnostic standard has been established well.^7^ However, some disadvantages such as long test time and the influence of sleep status limit its widespread application, and the consistency between nocturnal and sex-stimulated erections remains questionable.

Audiovisual sexual stimulation (AVSS) is an effective way to test psychogenic or reflex erection, which is considered the closest method of real sex-stimulated erection testing. Furthermore, the AVSS-induced erection test, as a simple, rapid, and objective diagnostic method, can be applied in the initial screening of ED in andrology outpatient clinics. Although the AVSS-induced erection test had been used in clinical practice for many years, the standardized operating procedure and diagnostic criteria still have not been well established. Some scholars believe that the diagnostic criteria of AVSS can refer to that of NPTR. However, the difference of erection modes determines that these 2 tests cannot share the same diagnostic criteria. The traditional AVSS test has some shortcomings, such as a low response rate, interference of environmental factors, and insufficient stimulation of the erotic video, which affects the accuracy of test. In order to reduce these influencing factors, some scholars ask the subjects to take phosphodiesterase type 5 (PDE5) inhibitor before AVSS test.^8^^,^^9^ However, the PDE5 inhibitor–induced erection does not reflect a true penile erection.

The development of virtual reality (VR) technology and its utility in clinical practice has attracted attention in the fields of biomedical therapy and public health.^10^ VR is a digital technology using computers to generate a virtual environment wherein sensory experiences (eg, visual, auditory, touch, and scent stimuli) prompt participants to observe and operate interactively.^11^ Previous studies had revealed the effectiveness of VR on relieving anxiety and depression.^12^ VR offers the opportunity to reduce physiological and psychological interference and increase visual and auditory feedback to the participants in clinical practice, which may enable the AVSS with VR (VR-AVSS) immersion test to be a more accurate detection than traditional and PDE5 inhibitor–induced AVSS modes. To the best of our knowledge, no previous studies have attempted to detect the penile erection parameters using VR immersion technology. The study aims to establish the standardized operating procedure and diagnostic criteria of the VR-AVSS test. We aim to address the following question: can VR-AVSS increase the erectile response rate of the AVSS test and improve the diagnostic accuracy of ED in male subjects?

## Methods

### Study design and population

All subjects 18 to 60 years of age were enrolled from 3 andrology centers including Renji Hospital, Shanghai Jiao Tong University School of Medicine, the Affiliated Hospital of Qingdao University and Northern Jiangsu People’s Hospital from June 2020 to March 2022. ED patients who had a complaint of erectile problems at least 6 months were enrolled from andrology outpatient in the hospitals. Control subjects recruited from the health screening center or fertility screening clinic of the hospitals. Before contacting potential participants, we searched recent medical records and clinical data for men who met our inclusion and exclusion criteria. Once we confirmed the status of general health, we contacted each person to assess his interest in participating and then start our screening and further evaluation. In order to ensure the perfect matching of the 2 groups, control patients received the same evaluation items with cases. Those in the ED and control populations must have had a stable relationship with a female partner and completed at least 1 sexual intercourse over the last 4 weeks. All subjects should have included detailed medical history records, comprehensive physical examination, psychological assessments such as Patient Health Questionnaire–9 and Generalized Anxiety Disorder–7, International Index of Erectile Function (IIEF-5), erectile hardness score (EHS), and self-reported erectile symptom questions. In the diagnostic test study, sample size was calculated by formula N_1_=$\frac{\left({Z}_{\mathrm{\alpha}}^2\right){S}_n\left(1-{S}_n\right)}{\Delta ^2}$ and N_2_=$\frac{\left({Z}_{\mathrm{\alpha}}^2\right){S}_p\left(1-{S}_p\right)}{\Delta ^2}$, α = 0.05, ${Z}_{\mathrm{\alpha}}$=1.96, where N_1_ represents the target disease sample size and N_2_ represents the control sample size. According to data from the literature, the expected sensitivity is 70% and the specificity is 80% in this study, and we set Δ = 0.1 as the admissible error. After the calculation, ED case sample size was N_1_ > 81, and N_2_ > 62 showed adequate power to achieve the expected effect.

This study had been registered on the Chinese Clinical Trial Registry (ChiCTR1800018577) https://www.chictr.org.cn/, and it received institutional review board approval (Renji Hospital, Shanghai, No. SK2020-089). The study was conducted in accordance with the ethical principles stated in the Declaration of Helsinki as revised in 2000, all subjects were provided written informed consent, and the participants who agreed to the study had signed the informed consent.

### Subjective erectile function assessment

All subjects were evaluated by IIEF-5 scores and EHS. Four self-reported erectile symptom questions were also provided to all subjects for erectile function evaluation, including question 1 (Q1), "do you have an erection when you watch a sex video?”; Q2, “do you have an erection when you sleep or get up in the morning?”; Q3, “do you have confidence in the erection?”; and Q4, “do you think the penile erection is hard enough?” The answer to every question was set as “yes” or “no,”

### VR-AVSS test

Subjects were arranged in a specific examination room with quiet environment, gentle light, and comfortable couch. The VR device (Neo2; PICO Technology) used in the test consists of head-mounted displays, body movement sensors, real-time videos, and advanced interface devices. The duration of the entire testing was set as 60 minutes, and the real-time videos were set as a landscape video for the calming-down phase in the first 10 minutes, an erotic video for the erecting phase in the middle 40 minutes, and a music video for the relaxing phase in the last 10 minutes. The erotic video including 4 short videos of 10 minutes each was edited from several commercial videotapes of heterosexual activity with sound including petting, foreplay, and intercourse. The order of erotic material segments in this erotic video was arranged according to the degree of sexual stimulation (from lowly to highly arousing). RigiScan Plus (Timm Medical Technologies) was used to record erectile parameters including radial rigidity, tumescence, and duration of erectile events. After the VR device was ready, a professional technician put the “base” and “tip” rings of RigiScan Plus around the root and distal of subject’s penile shaft, respectively. The RigiScan Plus would automatically and continuously record the penis circumference and hardness changes during the whole process. Matching with the videos, the RigiScan Plus recorded the baseline data in the first 10 minutes and then erectile parameters in the middle 40 minutes. In the event of equipment failure and/or ejaculation during the test, a second testing would be performed.

### The definition for RigiScan Plus test data

A negative response was no recording data in RigiScan software (excluding instrument fault), and tip and/or base rigidity displayed as 0%. A positive response was the tip and base average rigidity of more than 5% and a duration more than 5 minutes. Invalid data were defined as the tip and/or base average rigidity ≤5% and/or duration ≤5 minutes. According to European Association of Urology guidelines, a normal penile erection is indicated by the recording a tip rigidity of at least 60% with a duration more than 10 minutes in 1 erectile event of the total NPTR test,^3^ and a tip rigidity more than 60% considers that the penis can effectively complete penetration during sexual intercourse.^7^ Therefore, we set the holding time of the erection hardness as exceeding 60% as the effective erectile duration of the penis. The tip effective erectile duration (TEED) was the time of penile tip rigidity ≥60%. The base effective erectile duration (BEED) was the time of penile base rigidity ≥60%.

### Screening procedures and inclusion/exclusion criteria

**Figure 1:**
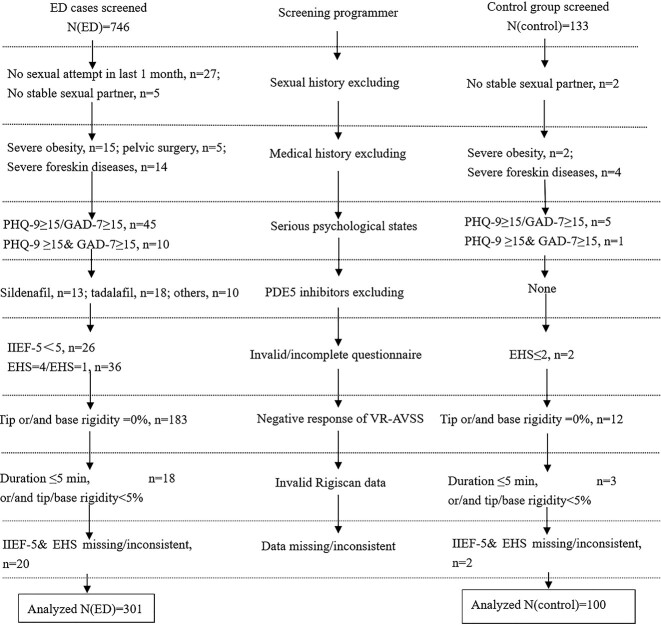
Screening flow chart of study participants. ED, erectile dysfunction; EHS, erectile hardness score; GAD-7, Generalized Anxiety Disorder–7; IIEF-5, 5-item International Index of Erectile Function; PDE5, phosphodiesterase type 5; PHQ-9: Patient Health Questionnaire–9; VR-AVSS, audiovisual sexual stimulation with virtual reality.

The detailed screening procedures and criteria are shown in Figure 1. First, all subjects had relatively fixed sexual partners and tried sexual intercourse within the last 4 weeks. Second, ED patients had an IIEF-5 score ≤21. Third, the control group had no complaint of erectile problems and an IIEF-5 score ≥22. Fourth, all subjects received a subjective erectile function assessment and VR-AVSS test. All subjects were excluded from undesirable sexual history, special medical history, serious psychological states, erection-related drug administration, invalid/incomplete questionnaire, negative VR-AVSS response, abnormal RigiScan data, and missing/inconsistent data, etc. Participants who had no sexual attempt in last 1 month or no stable sexual partner, severe obesity, pelvic surgery, severe foreskin diseases, and PDE5 inhibitor administration in last month were excluded. Men with Patient Health Questionnaire–9 score ≥15 and/or Generalized Anxiety Disorder–7 score ≥15 were excluded. Participants with key information missing and inconsistent data were also excluded. Invalid tests induced by any reasons such as negative response, invalid RigiScan data, instrument failure, and midway ejaculation were excluded.

### Statistical analysis

All analyses were conducted using SPSS 22.0 (IBM). Continuous variables with normal distribution indicated by mean ± SD were tested by 2 independent samples *t* tests, and continuous variables with non-normal distribution were indicated by median (interquartile range) and were tested by Mann-Whitney *U* test. The chi-square test was used for categorical variable data. The receiver-operating characteristic curve of best independent predictors was examined to identify a cutoff value in order to predict ED. *P* < .05 was considered statistically significant.

## Results

In all, 301 ED cases (31.07 ± 6.76 years of age) and 100 control patients (31.20 ± 6.15 years of age) were screened for analysis. The positive response rates of VR-AVSS in the ED and control groups were 75.5% (n = 563 of 746) and 90.9% (n = 121 of 133), respectively. The comparisons of variables between 2 groups are shown in Table 1. As expected, ED cases had significantly lower IIEF-5 scores than control patients. EHS and self-reported erectile reaction were lower in ED patients compared with the control patients. The erection hardness and maintenance time of subjects with positive VR-AVSS reaction were significantly different between the 2 groups (Table 1), especially the parameters such as tip average rigidity (TAR), TEED, base average rigidity (BAR), and BEED (*P* < .001). There were no significant differences in age, sexual video stimulation response (Q1) and perceived erection hardness (Q4) between the ED and control groups.

**Table 1:** Statistical characteristics of ED and control groups.

	ED group (n = 301)	Control group (n = 100)	*P* value
Age, y	31.07 ± 6.76	31.20 ± 6.15	.861
BMI, kg/m^2^	24.00 ± 3.31	25.04 ± 3.73	.011
IIEF-5 score	14.72 ± 3.65	22.72 ± 1.05	<.001
EHS	2.72 ± 0.45	3.30 ± 0.46	<.001
Q1 (yes)^a^	83.8	85.5	.714
Q2 (yes)^b^	69.5	82.9	.023
Q3 (yes)^c^	26.0	67.5	<.001
Q4 (yes)^d^	76.6	86.5	.070
Positive response rate	75.5	90.9	<.001
EED ≥5 min	31.7	83.0	<.001
EED ≥10 min	15.3	60.0	<.001
Tip duration of event, min	14.0 (10.0-23.5)	23.5 (12.0-33.0)	<.001
TAR, %	31.0 (23.0-40.5)	49.5 (38.0-61.5)	<.001
TEED, min	1.5 (0-4.0)	8.5 (2.5-19.5)	<.001
Δ C-tip, cm	2.46 ± 0.80	2.91 ± 0.79	<.001
Base duration of event, min	14.0 (10.0-23.5)	24.5 (12.0-34.0)	<.001
BAR, %	37.0 (30.5-46.0)	58.5 (46.5-65.5)	<.001
BEED, min	2.0 (0.5-6.0)	11.5 (6.5-26.5)	<.001
Δ C-base, cm	2.35 ± 0.63	2.83 ± 0.63	<.001

Values are mean ± SD, %, or median (interquartile range). For BEED, EED, and TEED, duration of rigidity was ≥60%.

Abbreviations: BAR, base average rigidity; BEED, base effective erectile duration; BMI, body mass index, ED, erectile dysfunction; EED, effective erectile duration; EHS, erectile hardness score; IIEF-5, 5-item International Index of Erectile Function Questionnaire Score; TAR, tip average rigidity; TEED, tip effective erectile duration; ΔC-base, changes in base erectile circumference; ΔC-tip, changes in tip erectile circumference.

^a”^Do you have an erection when you watch a sex video?”

^b^”Do you have an erection when you sleep or get up in the morning?”

^c^”Do you have confidence in the erection?”

^d^”Do you think the penile erection is hard enough?”

**Figure 2:**
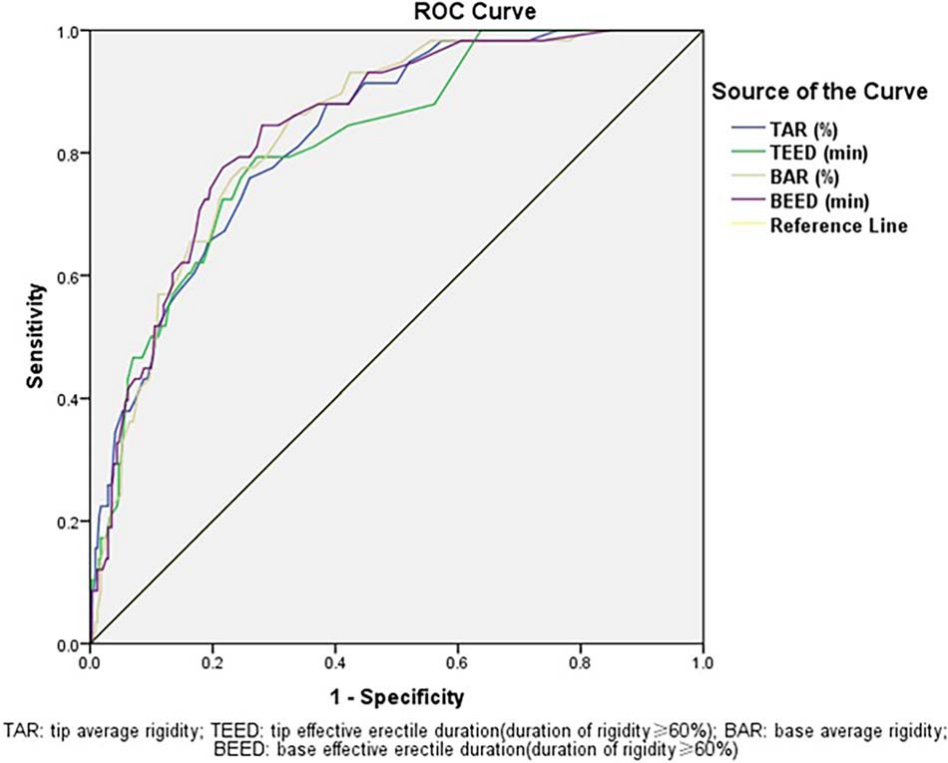
Receiver-operating characteristic curve (ROC) for tip average rigidity (TAR), tip effective erectile duration (TEED), base average rigidity (BAR), and base effective erectile duration (BEED) in erectile dysfunction (ED) and control groups.

According to the receiver-operating characteristic curve analysis, TAR, BAR, TEED, and BEED were the 4 most valuable parameters, and their area under the curve values were 0.829, 0.836 0.818, and 0.842 (Figure 2), respectively. TAR ≥40.5% (sensitivity: 77.6%; specificity: 70.2%), TEED for 4.75 minutes (sensitivity: 75.9%; specificity: 75.4%), BAR ≥48.5% (sensitivity: 77.6%; specificity: 75.1%), and BEED for 7.75 minutes (sensitivity: 79.3%; specificity: 75.7%) showed the highest diagnostic value for non-ED (Table 2).

**Table 2:** Diagnostic performance of TAR, TEED, BAR, and BEED.

Variable	AUC (95% CI)	*P* value	Cutoff point	Sensitivity	Specificity
TAR	0.829 (0.778-0.880)	<.001	40.5%	77.6%	70.2%
TEED	0.818 (0.762-0.873)	<.001	4.75 min	75.9%	75.4%
BAR	0.836 (0.788-0.885)	<.001	48.5%	77.6%	75.1%
BEED	0.842 (0.793-0.891)	<.001	7.75 min	79.3%	75.7%

For BEED and TEED, duration of rigidity was ≥60%.

Abbreviations: AUC, area under the curve; BAR, base average rigidity; BEED, base effective erectile duration; CI, confidence interval; TAR, tip average rigidity; TEED, tip effective erectile duration; ΔC-tip, changes in tip erectile circumference.

## Discussion

These findings in our study showed that the VR-AVSS test can effectively improve the value in distinguishing the ED and non-ED in male subjects. The indicators that TAR ≥40.5%, TEED more than 4.75 minutes, BAR ≥48.5%, and BEED more than 7.75 minutes suggested the best diagnostic values for excluding ED.

The AVSS-induced erection test, as a simple and rapid detection method, has been developed for many years in different clinical institutions. However, the traditional AVSS test may experience a low response rate or high false negative response rate induced by inadaptable testing surroundings, the subject’s psychological factors, and poor sexual arousal to a conventional monitor, which may limit its widespread application in clinical practice.^13^^,^^14^ In Kim et al’s study,^14^ the positive response rates (duration of average maximal rigidity ≥5 minutes) of conventional AVSS were 37% in normal men. Furthermore, only 3 (13.6%) of the 22 normal men sustained a penile rigidity of 70% or more for more than 5 minutes, and 10 (45.5%) had a rigidity of 40% or more. Park et al^15^ reported that 2-dimensional real-time images in AVSS enabled the subjects to acquire more sexual stimuli,, and the responsiveness rate was higher in using the special monitors than in using the conventional monitors, and they found the real-time AVSS during Doppler ultrasonography increased erectile quality (improvement of at least 1 grade) in 26 (65%) of 40 patients. Moon et al^16^ thought that 3-dimensional could provide a more realistic image and a more comfortable circumstance to subjects. Therefore, their study showed that 3-dimensional AVSS significantly increased the rate of normal erection responses compared with the conventional AVSS in control group (80% vs 33%).^16^ Recently, VR technology can generate a virtual environment combining visual, auditory, touch, and scent stimuli experiences, which will benefit psychotherapy and sexual dysfunction treatment.^17^ VR immersion is a powerful tool providing clients with new mode experiences, and many past studies had demonstrated that VR could improve erectile dysfunction and premature ejaculation by reducing psychological influence.^18–20^ In view of the various advantages, VR technology is considered as the optimal modality for the AVSS test. As expected in our study, the positive response rate of normal men (control group) induced by VR-AVSS significantly increased compared with that induced by other AVSS. The rates of average rigidity ≥5% with duration ≥5 minutes, EED ≥5 minutes, and EED ≥ 10 minutes in both tip and base were 90.9%, 83.0%, and 60.0%, respectively.

Although AVSS testing methods has been developed for many years in different clinical institutions, there is still no unified standardized operation process, especially the editing of erotic movie and viewing time. The past studies generally chose 2 or 3 commercial videotapes of heterosexual activity with sound for AVSS, and the film stimuli were varied in intensity from medium to highly arousing.^13^^,^^14^^,^^16^^,^^21^ In those studies, 2 suggested that each patient watched an erotic videotape edited from commercial films of heterosexual activity with sound for 30 minutes.^16^^,^^21^ The other 2 studies suggested that each subject watched the erotic movie for 20 minutes^14^ or even 15 minutes.^13^ Based on the operation process of previous research, we edited the video to be first a 10-minute landscape video for calming down, then a 40-minute erotic video composed of 4 short videos of 10 minutes each for sexual stimulation, and last a 10-minute music video for relaxing, and we found this playback mode could effectively reduce the impact of psychological factors and minimize detecting errors of the RigiScan Plus.

The diagnostic criteria of AVSS for ED are also still unclear. Most clinicians consider that the diagnostic criteria for normal erectile function of AVSS can refer to the criteria of NPTR that the rigidity of the penile tip is ≥60% with the duration of a single erection for more than 10 minutes.^3^ However, AVSS and NPRT are 2 completely different erection modes, and there may be greater bias if the 2 tests share the same reference standard. Kim et al’s^14^ AVSS data showed that the tip and base average rigidity of 25 control men were 36.55% ± 28.84% and 44.23% ± 31.76% in the fist day test, respectively. Wang et al^9^ suggested that a basal rigidity over 60% sustained for at least 8.75 minutes, average event rigidity of tip at least 43.5%, and base at least 50.5% would be the new normative evaluation criteria for the AVSS-RigiScan test. However, their test was based on conventional videos and oral PDE5 inhibitors, which may lead to low positive response rate and drug-induced false positive rate. Our multicenter and large sample data revealed that TAR ≥40.5%, TEED more than 4.75 minutes, BAR ≥48.5%, and BEED more than 7.75 minutes were the 4 key indicators to rule ED in subjects tested by VR-AVSS.

Different from similar previous studies, our data came from multicenter and prospective study included large sample ED and normal population, which provided a more reliable reference values to discriminate ED from normal erectile function. Moreover, we first applied VR immersion technology to establish the standard operation procedure for AVSS test, which could effectively reduce the interference of adverse factors and minimize the detecting errors. However, some disadvantages should also be considered. First, the videotape material used in this study were set in advance without choice for subjects. Therefore, some men might be insensitive to the erotic stimuli, or even feel disgusted by the videotape, which might cause low or negative erectile response. Second, the test data only included positive response subjects, so the true erectile status of men with a negative response to AVSS cannot be obtained. Last, VR-AVSS testing can effectively distinguish whether subjects have ED, but the etiological classification such as vascular or nonvascular ED remains unclear.

## Conclusion

VR-AVSS can significantly improve the erectile response rate of AVSS test in male subjects. The VR-AVSS test can effectively distinguish ED from subjects with positive erectile response to AVSS, and the diagnostic values for excluding ED are TAR ≥40.5%, TEED more than 4.75 minutes, BAR ≥48.5%, and BEED more than 7.75 minutes. More similar studies are needed to confirm the reliability of this results to better promote this technology in clinical practice of application.
